# Aerosol Transmission of Coronavirus and Influenza Virus of Animal Origin

**DOI:** 10.3389/fvets.2021.572012

**Published:** 2021-04-13

**Authors:** Jing Lv, Jing Gao, Bo Wu, Meiling Yao, Yudong Yang, Tongjie Chai, Ning Li

**Affiliations:** ^1^Shandong Provincial Key Laboratory of Animal Biotechnology and Disease Control and Prevention, Sino-German Cooperative Research Center for Zoonosis of Animal Origin Shandong Province, Shandong Provincial Engineering Technology Research Center of Animal Disease Control and Prevention, College of Animal Science and Veterinary Medicine, Shandong Agricultural University, Taian, China; ^2^Center for Disease Control and Prevention, Taian, China; ^3^Taian Central Hospital, Taian, China

**Keywords:** coronavirus, influenza virus, aerosol transmission, epidemiology, public health

## Abstract

Coronavirus disease 2019 (COVID-19) caused by severe acute respiratory syndrome coronavirus 2 (SARS-CoV-2) has caused great harm to global public health, resulting in a large number of infections among the population. However, the epidemiology of coronavirus has not been fully understood, especially the mechanism of aerosol transmission. Many respiratory viruses can spread via contact and droplet transmission, but increasing epidemiological data have shown that viral aerosol is an essential transmission route of coronavirus and influenza virus due to its ability to spread rapidly and high infectiousness. Aerosols have the characteristics of small particle size, long-time suspension and long-distance transmission, and easy access to the deep respiratory tract, leading to a high infection risk and posing a great threat to public health. In this review, the characteristics of viral aerosol generation, transmission, and infection as well as the current advances in the aerosol transmission of zoonotic coronavirus and influenza virus are summarized. The aim of the review is to strengthen the understanding of viral aerosol transmission and provide a scientific basis for the prevention and control of these diseases.

## Introduction

As human production and activities continue to infringe on the territory of wildlife by illegally capturing and consuming wild animals, the contact between humans and wild animals becomes more frequent, and the opportunities for unknown pathogenic microorganisms entering the human living environment have greatly increased. Coupled with changes in livestock and poultry feeding modes and adverse changes in climate and ecological environment in recent years, the prevalence and variation of pathogenic microorganisms, especially viruses, are accelerating, and the ability to spread across species is increasing, largely boosting the risk of zoonotic diseases in humans.

Coronavirus, as a main zoonotic virus, has caused three pandemics worldwide since the beginning of the twenty-first century. In 2003, severe acute respiratory syndrome (SARS) raged in China and 29 other countries and regions (1). In 2014, Middle East respiratory syndrome (MERS) spread through Middle East (2). The ongoing coronavirus disease 2019 (COVID-19) pandemic caused by severe acute respiratory syndrome coronavirus 2 (SARS-CoV-2) was first reported in Wuhan, China in 2019 (3–5), but as of now, the original source of SARS-CoV-2 is unclear and studies are continuing. COVID-19 is still rapidly spread around the world, and this disease is a rare major public health event in human history. As of December 29, 2020, there are over 79 million global confirmed cases and over 1.7 million deaths in 200 countries according to WHO COVID-19 situation report (6).

Many zoonotic diseases of animal origin, such as H5 and H7 subtype avian influenza, SARS, MERS, and other respiratory infectious diseases, can spread by airborne transmission, leading to rapid outbreaks within a short period of time (7–9). In March 2020, the National Health Commission of China successively released the sixth and seventh editions of the Protocol on Diagnosis and Treatment for COVID-19, in which it has stated the probability of aerosol transmission of SARS-CoV-2 under the conditions of long-term exposure to high-concentration aerosols in a relatively closed setting (10). The United States Centers for Disease Control and Prevention (CDC) also acknowledged that airborne transmission of SARS-CoV-2 could occur within enclosed space that had inadequate ventilation (11). These results reflect the continuous deepening of the understanding of COVID-19 transmission.

In this review, based on the research progress of aerosol transmission of zoonotic coronavirus and influenza virus, we summarized the characteristics of viral aerosol generation, transmission, and infection, as well as the experimental and clinical data of the human-to-human and animal-to-animal aerosol transmission. The aim of the review is to strengthen the understanding of viral aerosol transmission and provide a scientific basis for the development of rational protective measures for the prevention and control of COVID-19, including wearing masks, safety goggles, and face shields for healthcare workers (HCWs); strict isolation; and environmental disinfection measures.

## Virus Aerosol

### Definition and Characteristics of Virus Aerosol

Microbial aerosol refers to the stable colloidal system of microorganisms floating in air in the state of a single cell suspension or fused with dry solid or liquid particles. If the microorganism is a virus, it is called a virus aerosol (12, 13). The aerodynamic diameter of virus aerosol particle is generally considered to be <5 μm; virus aerosol transmission is fundamentally different from droplet transmission in terms of their colloidal and aerodynamic mechanisms. Droplets are often emitted when patients cough, sneeze, and talk, with larger particles >5 μm. However, large droplets can produce smaller aerosol particles through water evaporation; the latter can travel deeply into the respiratory tract (alveoli) and have a strong ability of penetration, increasing the pathogenicity of the enclosed pathogen (14). Although viruses suspended in aerosol do not have the conditions for growth, virus aerosol can act as an important spread carrier to increase the infection risk of diseases.

Virus aerosols have the following four characteristics: (1) Instability: virus aerosol is unstable from the beginning of its formation. Virus survival is affected by many factors, including viral structure, suspension medium, and environmental factors. Generally, viral activity decreases with time. (2) Irregular motion: viral particles undergo Brownian motion in the air, that is, they can move up and down, left and right, and back and forth in the three-dimensional space, leading to a longer suspension time and transmission distance. (3) Regenerability of deposited aerosols: once aerosol particles settled on the surface of environmental objects encounter airflow, vibration, or other mechanical forces, they can be raised again to produce a regenerated aerosol. (4) Extensive infection: viral aerosols can also invade the body through the conjunctiva, damaged skin, and digestive tract in addition to the respiratory tract (15). Therefore, in the treatment of patients with COVID-19, in addition to masks, additional protective measures including safety goggles and face shields are also necessary for HCWs to avoid contact with SARS-CoV-2. It is reported that the infectious dose of SARS-CoV-2 is believed to be low (1 × 10^2^−1 × 10^3^ particles) (16); we speculate this may partly be due to the structural and physiological characteristics of the respiratory tract, the amount of pathogens required to cause respiratory infections is significantly lower than that of the digestive tract, especially the emerging pathogen SARS-CoV-2, and humans and animals are more susceptible to it in the absence of immune resistance. The susceptibility of the respiratory tract and the frequency of human or animal exposure to virus aerosols determine the widespread infection.

### Generation of Virus Aerosol

**Figure 1:**
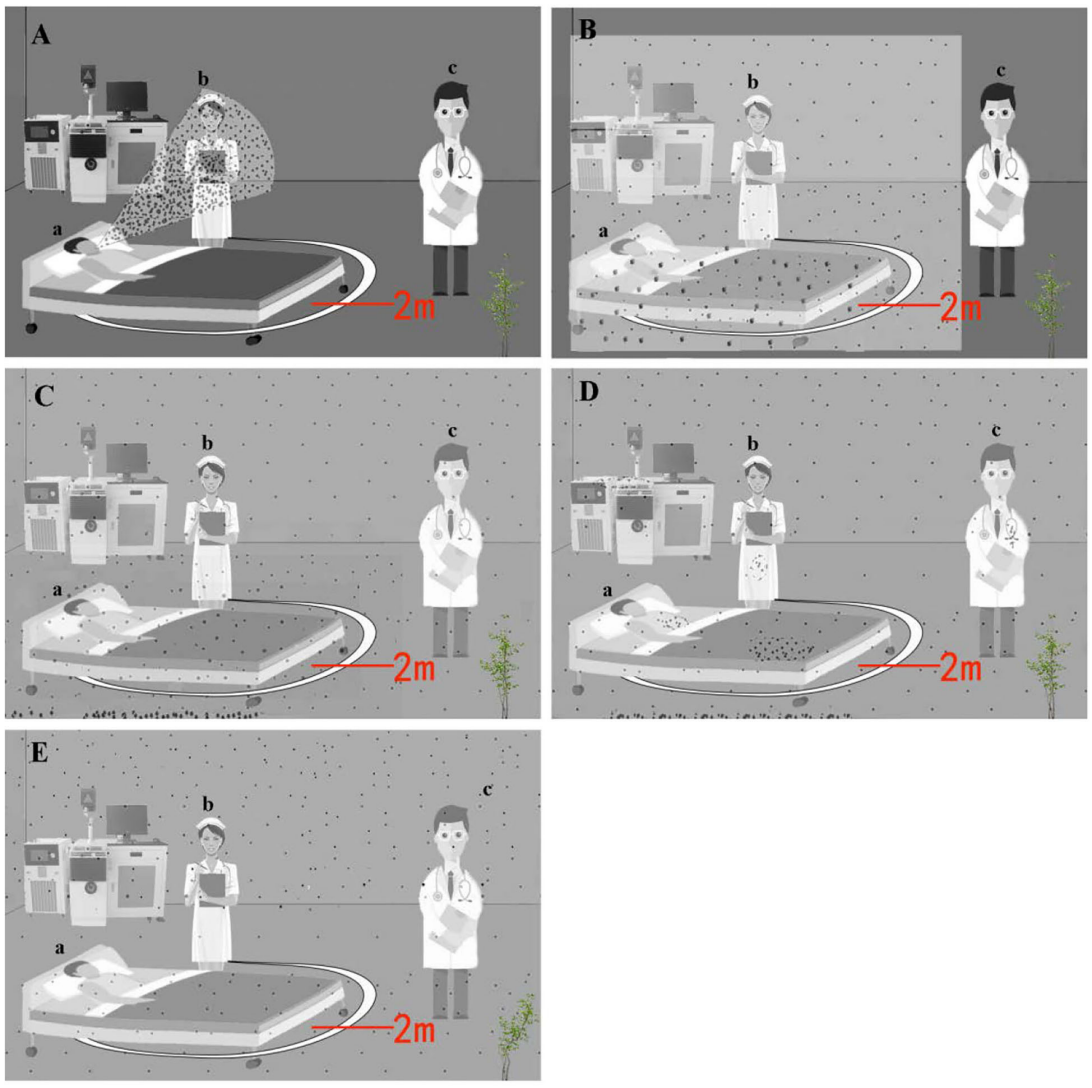
Schematic of aerosol emission, dispersion, and regeneration over time. The larger circles represent droplet, and the smaller circles represent aerosol particles. **(A)** At time = 0, microbiology aerosol and droplet are generated by patient (a); nurse (b) standing near him is exposed to and inhales a large amount of particles, but the doctor (c) has no exposure. **(B)** At time = 1, the aerosol is dispersing, and droplets are setting within 2 m; nurse inhales particles, and the doctor has no exposure. **(C)** At time = 2, the aerosol dispersed throughout the ward, and droplets have been deposited on the floor. Nurse and doctor inhale particles. **(D)** At time = 3, the aerosol particles have deposited on the bed, floor, equipment, etc. **(E)** At time = 4, airflow or aerosol-generating procedure (AGP) re-aerosolizes the tiny particles that have been deposited.

Virus aerosols in hospitals mainly come from the exhaled gas, respiratory secretions, and feces of patients. Studies have shown that a person can produce about 3,000 droplets per cough and as many as 40,000 droplets per sneeze (17). As shown in Figure 1, in an enclosed ward, aerosol plumes produced by patient (a) when coughing or sneezing contain many droplets and particles with different sizes (<5 or >5 μm). Nurse (b) who is close to the patient is exposed to the droplets and inhales them into the upper respiratory tract. The suspension time of a droplet is short, and its transmission distance is generally within 2 m, which is considered as a short-range transmission route, and at this moment, doctor (c) at a longer distance is not affected (Figure 1A). When the droplets are discharged into air, the larger droplets begin to settle on the surface of environmental objects (floor, walls, bed sheet, etc.) due to gravity, and meanwhile, the droplets begin to evaporate to form droplet nuclei with a diameter <5 μm, which is aerosol. Over time, the droplets will form more aerosols, and virus can be independently suspended or attached to the aerosol particles. Aerosols with small sizes and weights are diffused and suspended in the indoor air for a long time. These particles are too small to settle because of gravity; the smaller the aerosols, the further the distance they can travel (18).

In the example illustrated in Figure 1, the doctor has gone through the process from no exposure to virus aerosol to inhales a large amount of viral particles, which can enter the lower respiratory tract or alveoli, increasing the risk of inhalation infection (Figures 1B,C). With further extension of time, virus aerosol particles will gradually deposit on ward floor, walls, bed sheet, clothing, and equipment, and the concentration of aerosols in the air will be reduced (Figure 1D). However, the airflow caused by people activity can cause the re-suspension of the aerosols that have been deposited, which increases the infection risk (Figure 1E). Besides, the implementation of aerosol-generating procedure (AGP) operations in hospitals, such as tracheal intubation, increased the concentration of the virus aerosols and risk of SARS and MERS infection (19–21). Therefore, virus aerosols can be deposited, suspended, re-deposited, re-suspended, and continue to spread until the virus particles become inactive. As mentioned above, when people enter special circumstances containing virus aerosols without protection, especially confined spaces such as hospital intensive care units (ICU) and elevators, they are susceptible to COVID-19. This explains why one can get COVID-19 without direct contact with an infected patient. The simple mode of infection is from pathogen to environment and then to humans.

Microbial aerosols of animal origin mainly come from respiratory secretions and feces of diseased or infected livestock and poultry and their contaminated feed, drinking water, and litter undergoing weathering, corrosion, and abrasion (15).

### Survival of Virus Aerosol

The air environment does not provide the ideal conditions for the survival of pathogens. Viruses in aerosols are affected by various factors, and once inactivated, they generally lose their infectivity. The decay of virus in an aerosol usually includes two stages. In the first stage, about half of the virus is inactivated in the first few seconds after aerosol formation; in the second stage, the mortality slows down and is affected by the viral characteristics and environmental factors such as temperature, relative humidity (RH), ultraviolet rays, and electromagnetic radiation (17). To date, the survival time of the virus under different conditions has not been extensively investigated. One study shows that SARS coronavirus (SARS-CoV) can maintain its infectivity for 14 days at 4°C in hospital's sewer systems, but only for 2 days when the temperature rises to 20°C (22). SARS-CoV can survive for 2 weeks in a dry environment, but can only survive for 5 days at 22–25°C and 40–50% RH. Thereafter, the viral infectivity gradually decreases. At 38°C and 80–90% RH, the vitality of SARS-CoV gradually decreases after 24 h (23). MERS coronavirus (MERS-CoV) can survive in aerosols at 20°C and 40% RH for 10 min and on the surface of non-adsorbed objects for 8–48 h (24). SARS-CoV-2 remained viable in aerosols for 3 h with a reduction in infectious titer from 10^3.5^ to 10^2.7^ median tissue culture infective dose (TCID_50_)/l air at 21–23°C and 65% RH; it was stable on plastic and stainless steel and survived for 2–3 days at 21–23°C and 40% RH (25). In addition, the deposited virus aerosol is susceptible to secondary aerosolization caused by human or environmental factors.

These results above indicate that the virus aerosol can exist for a long time in a polluted environment. It should be noted that although most viruses are easily diluted after long-time and long-distance transmission, and some have even died, it is undeniable that aerosols can be transmitted as long-term vectors and cause infections of the viruses.

## Aerosol Transmission of Coronavirus and Influenza Virus

### Coronavirus

#### SARS-CoV-2

In the early stage of the COVID-19 outbreak, Huang et al. (26) clearly pointed out that this disease may be transmitted efficiently through human-to-human transmission, and it is strongly recommended to take measures to prevent airborne infections, such as the use of N95 masks. It is generally accepted that SARS-CoV-2 can spread via respiratory droplets during close contact and less commonly through contact with contaminated surfaces, but increasing evidences show that aerosols are strongly suspected to play a significant role in the rapid spread of COVID-19. A typical superspreading event reported in China involved 10 people from three families in the same air-conditioned restaurant. Authors found that the SARS-CoV-2 was able to propagate far enough to infect other members of the three families. The outbreak of COVID-19 cannot be explained by respiratory droplet transmission alone and aerosol transmission may be involved (27). Additionally, many studies have confirmed that SARS-CoV-2 can be detected in hospital air. Guo et al. (28) collected the air samples of ICU and general COVID-19 ward (GW) and found that 35% (14/40) and 12.5% (2/16) of samples tested positive for SARS-CoV-2 in ICU and GW, respectively. Moreover, air outlet swab samples also tested positive. Air samples in patient areas, medical staff areas, and public areas of two Wuhan hospitals were gathered during the COVID-19 outbreak in February and March 2020, and detection results showed that the highest concentration of SARS-CoV-2 RNA was 113 copies/m^3^ in ICU in patient areas; some medical staff areas initially had high virus concentrations in aerosols, and aerodynamic diameters of most SARS-CoV-2 were <2.5 μm (29).

Recently, Lednicky et al. (30) reported that viable SARS-CoV-2 was isolated from air samples gathered 2–4.8 m away from patients, with concentrations ranging from 6 to 74 TCID_50_ units/l of air. Patients with COVID-19 do not always cough and sneeze, so how is the viral aerosol produced? A recent study suggested that COVID-19 patients upon the onset of the disease were shown to emit about 10^5^ viruses per min (31). Given that a study demonstrated that SARS-CoV-2 remained infective with undiminished virion integrity for up to 16 h in aerosol droplets (32), aerosol transmission of SARS-CoV-2 is undoubtedly plausible (33). High concentrations of SARS-CoV-2 have been detected in patients' toilet without ventilation (29), and the virus can also be isolated from urine and feces (34). Therefore, the aerosol is very likely from patients' feces and urine in the toilet; COVID-19 may also be transmitted through fecal–oral route (35). SARS-CoV-2 is present not only in indoor air but also in outdoor environments. Researchers tested 34 samples of outdoor/airborne PM_10_ from Bergamo Province, Italy, and found that SARS-CoV-2 RNA can be present on outdoor particulate matter (36).

Since the outbreak of COVID-19, research on the relationship between SARS-CoV-2 and animal infection has been increasing. Several cases of human-to-animal transmission have been reported, such as dogs, cats, minks, etc., and they have different susceptibility to SARS-CoV-2 (37–39). The available animal models for SARS-CoV-2 have been extensively explored, including mice, cats, ferrets, and primates, which are of great significance for understanding the susceptibility of animal species to the SARS-CoV-2, the viral transmission route, and pathogenicity (40). Although the infectivity of aerosolized SARS-CoV-2 has not been explored, as mentioned above, we must pay close attention to the formation, survival, and propagation characteristics of SARS-CoV-2 aerosols, especially in hospital wards, confined spaces, and the surrounding outdoor environments.

#### SARS-CoV

SARS caused by SARS-CoV was first detected in Guangdong, China in 2002, and then quickly spread to Southeast Asia and other parts of the world. A large number of studies have found that SARS can spread through direct contact and droplets (41). However, clinical observation and analysis also suggest that SARS can be transmitted through aerosols. Studies have shown that 49% of SARS cases are related to hospitals, most likely caused by AGP for seriously ill patients (42–44). Tsai et al. (45) collected air samples near hospital beds (about 1 m) within 8 h after tracheal intubation and extubation for 11 SARS patients, and all samples tested positive for SARS-CoV. In a ward where a SARS patient lives, SARS-CoV is detected in air samples and swab samples on the surface of objects that are in frequent contact, although no live virus has been isolated in these samples (46, 47). Epidemiological investigations have pointed out that the infection in the Amoy Gardens in Hong Kong better shows that SARS is an opportunistic airborne transmission. Dense SARS-CoV aerosol plumes have been observed in contaminated sewers from index cases, which have spread to other buildings in the community, leading to 321 confirmed cases of infection. This outbreak of SARS also indicates that SARS-CoV aerosols can remain viable long enough to be transmitted to susceptible individual (48). Afterwards, hydraulic aerosol experiments, combined with epidemiological models, clearly indicate that the SARS outbreak in this community was transmitted by aerosol (9).

#### MERS-CoV

Since the first MERS case was confirmed in 2012 in Saudi Arabia, there have been a total of 858 patients who died from the infection and related complications in 27 countries (49). As a zoonotic virus, it has been reported that humans with MERS have been associated with direct or indirect contact with infected dromedary camels (50, 51). Human-to-human transmission of MERS has been documented, and it appears to be more frequent in health care settings than in the household (52, 53). Among the infection cases of MERS-CoV, 31% are related to hospitals (2, 42, 54). MERS-CoV can be detected from the bed sheets and medical and ventilation equipment contaminated by patients, and viruses can be isolated from some samples, indicating the presence of contact or contaminated fomites transmission (55). Of note, MERS-CoV has been detected and isolated from air samples in patient wards, toilets, and corridors, suggesting that this virus also has a great risk of airborne transmission (8).

In order to further understand the aerosol transmission of MERS-CoV, recently, several animal models of MERS-CoV aerosol infection have been established. In the mice infection model, hDPP4 transgenic mice were infected with MERS-CoV by an animal nose-only exposure device, and the results demonstrated that high viral loads were detected in lungs of MERS-CoV aerosol-infected mice, and they exhibited obvious diffuse interstitial pneumonia on day 7; moreover, the lung lesions more closely resembled those observed in humans (56). In primate model of MERS, African green monkeys were exposed to aerosolized MERS-CoV with different doses (10^3^,10^4^, and 10^5^ PFU), and all animals in the 10^5^ PFU group displayed overt respiratory disease signs, including chest congestion, rales, and wheezing, and a dose-dependent increase of respiratory disease signs was observed (57). As mentioned above, these experimental data confirm that MERS-CoV can spread through aerosols.

### Influenza A Virus

Influenza A virus is a well-known respiratory virus, including the 2009 swine influenza H1N1, seasonal influenza viruses, highly pathogenic avian influenza virus (HPAI) H5N1 and H7N9, and low pathogenic avian influenza virus (LPAI) H9N2, and many strains can be transmitted by aerosols (7, 58–61). In special circumstances, such as households with poor ventilation, the aerosols can be the main transmission route of influenza virus (62), which sufficiently indicates the key role of aerosols in influenza virus spread.

A large number of studies have proved that influenza virus aerosols can be detected in air samples from health care settings. Recently, Zhao et al. (63) collected 91 air samples daily from hospital outpatient hall, clinical laboratory, fever clinic, children's ward, and adult ward, during January and April 2018, and found that air samples collected from the children's ward, adult ward, and fever clinic were positive for airborne influenza viruses, including epidemic strain H1N1. Influenza virus can be detected in the air up to 3.7 m away from patients with the majority of viral RNA contained in aerosols (64). About 50% of the influenza viral RNA detected by Blachere et al. (65) in the emergency department of a hospital are from aerosols (aerodynamic diameter <4 μm). Quantitative analyses show that the highest load of the influenza virus is 13,426 median tissue culture infective dose (TCID_50_), and the median infective dose (EID_50_) of influenza virus aerosols (1–3 μm) in humans is about 0.6–3 TCID_50_ (66), which is significantly higher than the EID_50_ for humans.

Further studies have shown that influenza virus aerosols are released when influenza patients cough, sneeze, and breathe normally. Lindsley et al. (67) reported influenza viral RNA was detected in coughs. Twenty-three percent of the influenza RNA was contained in particles 1–4 μm in aerodynamic diameter, and 42% was in particles <1 μm. Viable influenza virus was detected in cough aerosols from 2 of 21 subjects with influenza. Fabian et al. (68) reported that, among 12 influenza patients, the virus was directly detected in the exhaled breath of four patients through qPCR. The generation rate can reach as high as 20 RNA copies per minute. Further analysis indicates that exhaling 20 RNA copies per minute is equivalent to excreting 4 TCID_50_ viruses per hour, suggesting that one is also at risk of infection during normal conversation with an influenza patient. Furthermore, aerosol particles emitted by patients contained viable influenza virus (69, 70). In the environment of a poultry farm, Lv et al. (15) collected 18 air samples from 6 chicken farms in different areas of Shandong Province and found that the concentration of H9 subtype influenza aerosol is about 1.25 × 10^4^-6.92 × 10^4^ copies/m^3^ air. These results indicate that influenza virus can naturally form aerosols, and more importantly, aerosols act as an important carrier for viral transmission.

In addition to the epidemiologic evidences of influenza viruses' aerosol transmission, mammals and poultry are used to perform experimental infection studies of influenza virus. Influenza infection has been documented by aerosol exposure in the guinea pig model. In this animal model, Lowen et al. (71) demonstrated that influenza virus was transmitted from infected guinea pigs to non-infected guinea pigs housed in an adjacent cage separated by 91 cm. Subsequently, they described a stronger experimental evidence for influenza aerosol transmission, and they documented the instance of transmission with the distance between cages increasing to 107 cm (72). Additionally, transmission of HPAI H5N1 by aerosols from geese to quails has been demonstrated in experimental infections (73). Airborne transmission of HPAI viruses can occur among poultry and from poultry to humans who are exposed to infected poultry (61). HPAI H9N2 infection experiment of SPF chickens via aerosol and nasal and digestive tract infusion routes was conducted by Yao et al. (74), and it was found that the aerosol route requires the lowest dose of the virus. The viruses required for nasal and digestive tract routes are 2 and 113 times those for aerosol, respectively, indicating that the virus aerosol infection efficiency is very high.

Notably, not all influenza virus strains can form aerosols, and different strains differ significantly in their capacity for aerosol transmission (72). Further study confirmed that amino acid mutations (D368E, S370L, E313K, and G381D) in the neuraminidase gene of H9N2 subtype AIV can significantly affect their aerosol transmission and viral replication ability in the respiratory tract (60). The H5N1 strain (Indonesia/5/2005) acquired mutations [four amino acid substitutions in the hemagglutinin (HA) gene and one in the polymerase complex protein basic polymerase 2 gene] during passage in ferrets, and ultimately, it was able to spread via aerosols among ferrets (7). The molecular mechanism of HA amino acid mutation that enables the H5N1 to transmit in ferrets by aerosols is the change of the complex structures of viral protein and the decreased affinities for receptors (75). These epidemiologic observations and infection experiments strongly support the view that influenza infections can occur via the aerosol route (76).

## Discussion

With the outbreak of COVID-19, the aerosol transmission of pathogens has once again become a hot topic. In this article, we mainly reviewed the aerosol transmission of several important zoonotic viruses, including influenza virus and coronavirus. Through direct examination of patients' exhaled breath and air samples in hospital wards, influenza virus is found in the air, which can infect the susceptible population. More importantly, in the household environment, the main transmission route of influenza virus among family members is aerosols (62). SARS-CoV, SARS-CoV-2, and MERS-CoV are severe respiratory pathogens, which are generally believed to be transmitted through contact and droplets. Because in practice it is very difficult to completely rule out contributions of a given mode of transmission, the relative contribution of each mode is usually difficult to establish by epidemiologic studies alone (76). Airborne transmission is the main route for efficient transmission between humans (59). However, there is an argument that viral RNA detectable in air samples is circumstantial and correlated with disease state rather than direct evidence of causality; therefore, it is very necessary to establish an animal model of viral aerosol infection for actual experimental demonstration. All in all, although SARS-CoV-2 has not yet been used to conduct aerosol generation and infection experiments, experimental data, epidemiological investigation, and protective measures against airborne infection have proven the likelihood of aerosol transmission of SARS-CoV-2. Therefore, measures such as protective clothing, masks, safety goggles, face shields, room ventilation, and strict disinfection are necessary to cut off the chain of infection.

The crucial role of aerosols in the transmission of respiratory infectious diseases cannot be denied, although not all pathogens solely depend on aerosol transmission. Especially under special environmental conditions, there is no reason to underestimate the importance of viral aerosol transmission. Studies should be focused on clarifying the mechanism of viral aerosol generation and survival and fully understanding the transmission mechanism of infectious diseases, so as to formulate prevention and control strategies for the COVID-19 pandemic and other newly emerging pathogens.

## Author Contributions

JL and JG wrote the manuscript. JL, BW, and MY performed the relative experiments of H9N2 AIV aerosol. NL and TC designed, revised, and approved the review. All authors contributed to the article and approved the submitted version.

## Conflict of Interest

The authors declare that the research was conducted in the absence of any commercial or financial relationships that could be construed as a potential conflict of interest.
